# Prolactinomas, Cushing's disease and acromegaly: debating the role of medical therapy for secretory pituitary adenomas

**DOI:** 10.1186/1472-6823-10-10

**Published:** 2010-05-17

**Authors:** Beverly MK Biller, Annamaria Colao, Stephan Petersenn, Vivien S Bonert, Marco Boscaro

**Affiliations:** 1 Neuroendocrine Clinical Center, Massachusetts General Hospital, Boston, USA; 2 Dipartimento di Endocrinologia ed Oncologia Molecolare e Clinica, Università degli Studi di Napoli Federico II, Napoli, Italy; 3 Division of Endocrinology, University of Duisburg-Essen, Essen, Germany; 4 Department of Medicine, Cedars-Sinai Medical Center, David Geffen School of Medicine, University of California, Los Angeles, California, USA; 5 Division of Endocrinology, Institute of Internal Medicine, Polytechnic University of Marche, Ancona, Italy

## Abstract

Pituitary adenomas are associated with a variety of clinical manifestations resulting from excessive hormone secretion and tumor mass effects, and require a multidisciplinary management approach. This article discusses the treatment modalities for the management of patients with a prolactinoma, Cushing's disease and acromegaly, and summarizes the options for medical therapy in these patients.

First-line treatment of prolactinomas is pharmacotherapy with dopamine agonists; recent reports of cardiac valve abnormalities associated with this class of medication in Parkinson's disease has prompted study in hyperprolactinemic populations. Patients with resistance to dopamine agonists may require other treatment.

First-line treatment of Cushing's disease is pituitary surgery by a surgeon with experience in this condition. Current medical options for Cushing's disease block adrenal cortisol production, but do not treat the underlying disease. Pituitary-directed medical therapies are now being explored. In several small studies, the dopamine agonist cabergoline normalized urinary free cortisol in some patients. The multi-receptor targeted somatostatin analogue pasireotide (SOM230) shows promise as a pituitary-directed medical therapy in Cushing's disease; further studies will determine its efficacy and safety. Radiation therapy, with medical adrenal blockade while awaiting the effects of radiation, and bilateral adrenalectomy remain standard treatment options for patients not cured with pituitary surgery.

In patients with acromegaly, surgery remains the first-line treatment option when the tumor is likely to be completely resected, or for debulking, especially when the tumor is compressing neurovisual structures. Primary therapy with somatostatin analogues has been used in some patients with large extrasellar tumors not amenable to surgical cure, patients at high surgical risk and patients who decline surgery. Pegvisomant is indicated in patients who have not responded to surgery and other medical therapy, although there are regional differences in when it is prescribed.

In conclusion, the treatment of patients with pituitary adenomas requires a multidisciplinary approach. Dopamine agonists are an effective first-line medical therapy in most patients with a prolactinoma, and somatostatin analogues can be used as first-line therapy in selected patients with acromegaly. Current medical therapies for Cushing's disease primarily focus on adrenal blockade of cortisol production, although pasireotide and cabergoline show promise as pituitary-directed medical therapy for Cushing's disease; further long-term evaluation of efficacy and safety is important.

## Review

Pituitary adenomas are classified according to the characteristic cell type from which the adenoma is derived, and include prolactinomas, somatotroph, corticotroph, gonadotroph, thyrotroph and null-cell (nonsecretory) adenomas. Morbidity associated with pituitary adenomas results from mass effects of the tumor and/or the effects of excessive hormone secretion [1]. Estimates of the prevalence of pituitary adenomas in the general population vary greatly. Autopsy and radiological series have suggested that small pituitary tumors may be present in as many as one in every six people, although these series include a large proportion of lesions that are not clinically significant (incidentalomas) [2]. The prevalence of clinically relevant pituitary adenomas in a recent Belgian study was found to be 1 per 1064 individuals [3], which is 3.5-5 times higher than previously reported estimates of clinically relevant adenomas; prolactinomas were most common (66%), followed by null-cell adenomas (14.7%), somatotroph adenomas (13.2%) and corticotroph adenomas (5.9%) [3].

The aim of this article is to review the treatment modalities for the management of patients with the three most frequent hormone-producing tumors: prolactinomas, corticotroph adenomas (Cushing's disease) and somatotroph adenomas (acromegaly), and to summarize the medical therapy available and under investigation in these patients.

### Management of prolactinomas

Prolactinomas are benign prolactin-producing pituitary adenomas. In women, the symptoms of hyperprolactinemia include infertility, menstrual irregularities, amenorrhea, galactorrhea, reduced libido and vaginal dryness, while in men they include impotence, reduced libido and infertility. If the tumor is large (a tumor with a diameter of >1 cm is classified as a macroadenoma), visual field defects and headache may be the presenting features. Therefore, patients requiring therapy for prolactinomas can be divided into two groups: those requiring therapy for the symptoms of hyperprolactinemia and those requiring treatment for the effects of tumor burden [4], although some patients have both hyperprolactinemia and mass effects, while others may have neither characteristic and may not need treatment.

The goal of treatment for most patients with prolactinomas is restoration of normal prolactin secretion if there are symptoms directly caused by prolactin excess, and prevention of hormonal/metabolic consequences (eg hypogonadism and osteoporosis) [5]. In patients with large tumors compromising the neurovisual apparatus (which represents the minority of cases), a decrease in tumor size is also a goal of treatment.

Current therapeutic options for patients with prolactinomas include observation, medical therapy with dopamine agonists (eg bromocriptine, cabergoline), transsphenoidal or transcranial surgery and conventional or stereotactic radiotherapy.

Surgery is generally reserved for patients who do not respond to medical therapy, patients with unstable pituitary hemorrhage or pregnant patients with progressive tumor enlargement not responding to dopamine agonist therapy. It may also be used in patients with contraindications to dopamine agonist use, such as those patients requiring psychiatric medications that work through the dopaminergic system. Transsphenoidal surgery is the surgical method of choice, with success rates in centers with experienced neurosurgeons (based on normalization of prolactin levels) of approximately 75% in patients with microprolactinomas and 34% in patients with macroprolactinomas [4]. Transcranial surgery is rarely performed, and is indicated in patients with tumors inaccessible via the transsphenoidal route. Radiotherapy is typically reserved for patients with large tumors jeopardizing adjacent structures who have not responded to medical or surgical treatment. Normalization of prolactin levels after radiotherapy is achieved in about one-third of patients, and the main complication is radiotherapy-induced hypopituitarism [4].

#### Medical treatment of prolactinomas

Prolactin secretion is regulated by the inhibitory signal of dopamine, acting via the dopamine D_2 _receptor [4]. Dopamine agonists have been used for the treatment of prolactinomas for over 25 years, and are widely considered the first-line therapy in patients with prolactinomas. Bromocriptine was the first dopamine agonist available for the treatment of prolactinomas, but others with a longer half-life, such as pergolide, quinagolide, and cabergoline, have since become available (although pergolide and quinagolide are not available in the US). The most extensive experience has been with bromocriptine, although cabergoline is now widely used. In most patients with prolactinomas, treatment with dopamine agonists normalizes prolactin levels, restores gonadal function and fertility, and reduces the size of the tumor. In patients with microprolactinomas, macroprolactinomas or idiopathic hyperprolactinemia, normal prolactin secretion was achieved in 76% of 997 patients treated with bromocriptine, 87% of 98 patients who received pergolide, and 89% of 612 cabergoline-treated patients [6]. With respect to the efficacy of this class of medication on tumor reduction in patients with macroprolactinomas, cabergoline appears to be the most effective, although there have not been direct comparisons. In three separate studies in patients with macroprolactinomas who received 12-27 months of first-line therapy with dopamine agonists [7-9], tumor reduction of more than 50% was seen in 64%, 86% and 96% of patients treated with bromocriptine, pergolide and cabergoline, respectively (Table 1).

**Table 1:** Effects of first-line therapy with bromocriptine, pergolide or cabergoline on tumor size and prolactin levels in patients with macroprolactinomas [7-9]

	Bromocriptine (n = 27)	Pergolide (n = 22)	Cabergoline (n = 26)
Baseline prolactin (μg/L)	2260	2938	1013
Normalized prolactin (% of patients)	66	68	100
≥50% tumor reduction (% of patients)	64	86	96
Duration of treatment (months)	12	27	24

Although first-line therapy with dopamine agonists is widely considered the preferred first-line treatment in patients with prolactinomas, a definitive cure has been considered possible only with surgery or, rarely, surgery plus radiotherapy. In patients successfully treated with dopamine agonists as primary therapy, it has been unclear if the withdrawal of dopamine agonists is effective and safe [10]. However, Colao *et al *have recently evaluated the long-term effects of dopamine-agonist withdrawal in patients who received cabergoline as first-line therapy, and showed that cabergoline can be safely withdrawn in patients who have achieved normal prolactin levels and have no evidence of residual tumor [11]. Patients in this study had nontumoral hyperprolactinemia (n = 25), microprolactinomas (n = 105) or macroprolactinomas (n = 75) at baseline, and had been receiving primary therapy with cabergoline 0.25-3.5 mg/week for 24-75 weeks. Withdrawal of cabergoline was considered if patients had normal prolactin levels and either no tumor visible by MRI or at least 50% reduction in tumor volume. The Kaplan-Meier estimated rate of recurrence of hyperprolactinemia 5 years after cabergoline withdrawal was low in patients who had a tumor at baseline and achieved tumor disappearance, and the recurrence rate did not differ significantly between patients with nontumoral hyperprolactinemia (24%), microprolactinomas (26.2%) or macroprolactinomas (32.6%). Recurrence of hyperprolactinemia was higher in patients still presenting small remnant tumors on MRI (41.5% in micro- and 77.5% in macroprolactinomas). However, in no case of biochemical recurrence did the tumor regrow or symptoms reappear; therapy was reinitiated when prolactin rose [11]. An additional analysis of estimated rates of recurrence at 7 years showed remission rates in patients with small remnant tumors was much lower in patients with macroprolactinomas than in patients with microprolactinomas (0% vs 20%; *P *< 0.0001) [4]. Furthermore, Cox regression analysis determined that tumor diameter on withdrawal of cabergoline is the major determinant of recurrent hyperprolactinemia [4]. These results show that cabergoline can be safely withdrawn in patients with normalized prolactin levels and no evidence of tumor, although patients should be closely monitored upon withdrawal of therapy, particularly those patients with macroprolactinomas in whom renewed growth of the tumor may compromise vision. Whether "drug holidays" should be advised in patients with a substantial decrease in tumor size, given the recent concern about the effects of cabergoline on cardiac values, is not yet clear.

#### Safety of dopamine agonists

The most common adverse effects associated with dopamine agonists are nausea and vomiting (~30%), headache (~30%), and dizziness (~25%). Although the type of adverse events associated with different dopamine agonists are similar, the incidence of these events is lower with cabergoline and generally less severe than those associated with bromocriptine or pergolide. Withdrawal of cabergoline therapy because of adverse events is reported in less than 3% of patients, compared with about 12% of patients treated with bromocriptine [12].

Of concern are recent reports describing the occurrence of valvular insufficiency in patients treated with cabergoline or pergolide for Parkinson's disease. These studies showed that the use of cabergoline and pergolide in patients with Parkinson's disease is associated with an approximately 5-fold increased risk of newly diagnosed cardiac valve regurgitation [13,14].

There have been nine recently published studies that evaluated the prevalence of cardiac valve regurgitation in a total of 608 patients with prolactinomas who received long-term cabergoline treatment [15-23]. Eight of the nine studies showed no evidence of clinically relevant valvular regurgitation after prolonged cabergoline treatment [16-23], although three of the eight studies did show an increased prevalence of mild tricuspid regurgitation in cabergoline-treated patients [16,20,23]. However, a significant increase in moderate tricuspid regurgitation was seen with cabergoline treatment compared with controls in an observational, case-control study by Colao *et al*. [15] that compared cardiac parameters in 50 patients with prolactinomas treated with long-term cabergoline therapy (median 6.2 years; range 1.1-10.7 years), 50 control patients and 20 patients with *de novo *prolactinomas. Chronic cabergoline treatment did not induce regurgitation of mitral, aortic or pulmonic valves, but an increased prevalence of moderate tricuspid regurgitation was seen in treated patients compared with controls (54% vs 18%; *P *< 0.0001) and *de novo *patients (54% vs 0%; *P *< 0.001). Tricuspid regurgitation was twice as frequent in patients with a cumulative cabergoline dose of more than 280 mg than in patients treated with lower cumulative cabergoline doses [15].

A recent meta-analysis [24] that pooled results from six of the studies [15,16,19-22] showed that patients treated with cabergoline were at increased risk of tricuspid regurgitation than control patients, with a prevalence ratio of 1.40 [95% CI 1.17-1.67].

Results from these studies do not provide a definitive answer, and long-term investigation of this issue will be important. Some centers suggest echocardiography follow-up studies in patients with prolactinomas treated with higher doses of cabergoline or other ergot-derived drugs.

#### Resistance to dopamine agonists

Although dopamine agonists are an effective treatment for most patients with prolactinomas, some patients do not achieve a satisfactory response. Resistance to dopamine agonists can be defined as a failure to achieve normalization of prolactin levels or no reduction in tumor size after 12-24 months of bromocriptine 15 mg/day or cabergoline 0.5 mg/day [6,25-27]; most cases of resistance to dopamine agonists can be considered partial resistance [28]. Approximately 24% and 11% of patients demonstrate resistance to bromocriptine and cabergoline, respectively [4]. In patients demonstrating possible resistance to a particular dopamine agonist, increasing the dose of that agent, as tolerated, may be effective as long as there is a continuing step-wise decrease in prolactin level with each step-wise increase in dose. Switching to another agent may be effective, but patients with resistant adenomas will usually require surgery, with or without radiotherapy, if control of a large lesion is needed.

A possible future medical therapy for patients with prolactinomas resistant to dopamine agonists is the multi-receptor targeted somatostatin analogue pasireotide (SOM230). In contrast to GH-secreting pituitary adenomas which express predominantly somatostatin receptor subtypes sst_2 _and sst_5_, prolactinomas express primarily sst_1 _and sst_5_. Unlike octreotide, which has high affinity for sst_2_, pasireotide has a multi-receptor binding profile, with affinity for sst_1,2,3 _and sst_5_. An *in vitro *study in prolactinomas cells has demonstrated a stronger inhibition of prolactin with pasireotide than with octreotide, and that the inhibition of prolactin release was related to the expression level of sst_5 _[29]. Studies with pasireotide have not yet been conducted in patients with prolactinomas.

Because prolactinomas express both D_2 _and sst_5 _receptors, there is a rationale for the use of chimeric D_2_/sst_5 _agonists such as BIM23A760, which has high sst_2 _and D_2 _activity and moderate sst_5 _activity [30,31]. A recent *in vitro *study of primary cultures of ten prolactinomas (six responsive to dopamine agonists and four resistant to dopamine agonists) showed that BIM 23A760 and cabergoline produced a similar partial inhibition of prolactin secretion [32].

#### Conclusions

First-line treatment of patients with prolactinomas is pharmacotherapy with dopamine agonists. In some centers with expert pituitary surgeons, selected patients with microprolactinomas may be offered surgery based on the high likelihood of definitive cure, although there are regional differences in how frequently surgery is advised. Whether an increased risk for tricuspid regurgitation associated with cabergoline, especially at higher doses and with longer treatment periods, should be investigated carefully. Patients fully responsive to dopamine agonists who have achieved tumor disappearance can be considered for withdrawal from the drug with careful follow up. A small subset of patients with macroprolactinomas that are less responsive to dopamine agonists may require more aggressive treatment with surgery and/or radiotherapy if tumor location or growth rate raises concerns about compromise of adjacent neurological structures.

### Management of Cushing's disease

Cushing's disease is caused by excessive secretion of adrenocorticotrophic hormone (ACTH) by a pituitary corticotroph adenoma resulting in enhanced cortisol secretion from the adrenal glands. Features of hypercortisolism include weight gain, severe fatigue and muscle weakness, high blood pressure, depression, cognitive impairment, purplish skin striae, hyperpigmentation, loss of libido, impaired glucose metabolism, hirsutism, acne, and menstrual disorders [33,34]. Chronic hypercortisolism is associated with an increased incidence of systemic arterial hypertension, diabetes mellitus, central obesity, hyperlipidemia and hypercoagulability [33]. Indeed, 20-50% of the patients with Cushing's disease have overt diabetes mellitus, whereas impaired glucose tolerance is present in 30-60% of patients [35]. Cushing's disease is associated with increased mortality, largely because of cardiovascular complications, including coronary heart disease, congestive heart disease and cerebrovascular events [36].

Treatment goals in Cushing's disease include the reversal of clinical features, the normalization of cortisol levels with minimal morbidity while preserving pituitary function, and long-term disease control without recurrence. In the small number of patients with macroadenomas, removal of the tumor mass is another treatment goal. The initial treatment of choice for Cushing's disease is generally selective pituitary adenomectomy by an experienced, dedicated pituitary surgeon. Second-line therapy includes more radical surgery, radiation therapy, including stereotactic radiosurgery, medical therapy and bilateral adrenalectomy [37,38] Cure rates in patients with Cushing's disease 10 years after pituitary surgery or radiotherapy are approximately 77% and 55%, respectively [39]. Although bilateral adrenalectomy is a definitive treatment providing immediate control of hypercortisolism, patients require lifelong glucocorticoid and mineralocorticoid replacement therapy, and up to 25% of patients develop Nelson's syndrome or at least regrowth of the underlying pituitary adenoma [40]. Nelson's syndrome is the aggressive growth of a pituitary corticotroph adenoma after bilateral adrenalectomy, and is associated with symptoms such as skin hyperpigmentation, headache and visual impairment, which arise due to the mass effect of the tumor and increased ACTH secretion [41]. Close monitoring by regular MRI scans and measurement of plasma ACTH levels should be undertaken to detect the occurrence of corticotroph tumor progression. Early detection offers the possibility of cure by surgery (microadenoma) or radiotherapy (invasive adenomas).

Currently, there are no curative medical therapies available for Cushing's disease. The most commonly used agents reduce cortisol levels via inhibition of steroidogenesis in the adrenal glands and, thus, do not target the underlying cause of the disease. Adrenal-directed therapy (eg ketoconazole and metyrapone) is generally used in the preoperative preparation of patients with severe disease, or in patients awaiting a response to radiation therapy.

#### Adrenal-directed therapy

Ketoconazole and metyrapone are the most commonly used steroidogenesis inhibitors for the treatment of Cushing's disease. As reviewed by Miller and Crapo [42], 80% of 72 patients with Cushing's disease treated with ketoconazole 600-1200 mg/day achieved normalization of urinary free cortisol (UFC) levels. A recent retrospective analysis of the long-term hormonal effects and tolerance of ketoconazole has been conduced in 38 patients with Cushing's disease, with a mean follow-up of 23 months. The data demonstrate good tolerability and effective control, particularly in patients for whom surgery is contraindicated, or delayed during the investigation of an occult adenoma [43].

In 53 patients with Cushing's disease treated for 1-16 weeks with metyrapone 750-6000 mg/day, 75% achieved normalization of mean daily plasma cortisol [44]. However, there is little prospective data on the long-term use of steroidogenesis inhibitors, and adverse effects limit their therapeutic use in some patients. Adverse effects associated with ketoconazole include abnormal liver tests (15%), gynecomastia (13%), gastrointestinal effects (8%), edema (6%), and skin rash (2%) [42], whereas adverse events associated with metyrapone doses >2 g/day include dizziness and ataxia (15%), nausea (5%), skin rash (4%), edema (8%) and hirsutism (70%) [44].

Mitotane and etomidate are also sometimes used in the treatment of Cushing's disease. Mitotane is a derivative of dichlorodipehyldichlororoethane (DDD) that specifically inhibits cells of the adrenal cortex. This adrenolytic action may prove effective in the long term suppression of hypercortisolism in the majority of patients with ACTH-dependent Cushing's syndrome [45,46]. Its mechanism of action also prevents the risk of escape phenomenon in response to the ACTH rise that occurs in Cushing's disease when plasma cortisol is decreased. However, its onset of action is slow (weeks or months), and the adverse effects associated with mitotane therapy (mainly digestive and neurological) require careful monitoring of drug levels, and it is routinely used in only a few centers. Etomidate is a non-opioid anesthetic that also induces adrenocortical suppression as one of its main side effects. In situations where rapid control of cortisol levels is required and oral therapy is problematic, iv etomidate therapy may be considered [47-49].

#### Pituitary-directed therapy

Pituitary-directed medical therapy targets the underlying cause of the disease, i.e. the ACTH-secreting tumor. A medical therapy that reverses tumor growth and lowers ACTH secretion to normal levels, thus normalizing the amount of cortisol being produced by the adrenal glands, would be a valuable therapeutic option for the management of Cushing's disease. Several pituitary-directed agents are under evaluation for the treatment of Cushing's disease, including peroxisome proliferator-activated receptor-gamma (PPAR-γ) agonists such as rosiglitazone and pioglitazone, dopamine agonists such as bromocriptine and cabergoline, and somatostatin analogues such as the investigational agent pasireotide.

PPAR-γ is a member of the nuclear receptor super-family and functions as a transcription factor. PPAR-γ ligands have been shown to inhibit cell growth in a number of tumor types [50], and rosiglitazone has demonstrated *in vitro *antiproliferative and apoptotic effects in corticotroph cells [51]. However, evaluation of normal pituitary tissue and pituitary tumors has shown poor expression of PPAR-γ receptor in human pituitary tissue, no detection of a specific abnormality in PPAR-γ expression in corticotroph tumors, poor immunocytochemical expression in both normal pituitary and pituitary adenomas, with only weak cytoplasmic staining [52]. In addition, the antiproliferative effect of rosiglitazone was shown only at very high doses and these were not blocked by a specific PPAR-γ antagonist. In two small clinical studies of rosiglitazone in patients with Cushing's disease, normalization of UFC levels was achieved in 37.5% of patients (9/24), whereas in one study of pioglitazone in five patients with Cushing's disease, no patients achieved UFC normalization [50,53,54]. More data are required not only for efficacy, but also to address the long-term safety of rosiglitazone in these patients, particularly in light of the recent report that rosiglitazone is associated with a significant increase in the risk of myocardial infarction in patients with type 2 diabetes mellitus [55].

The dopamine D_2 _receptor is expressed in approximately 75% of corticotroph adenomas [56]. Both bromocriptine and cabergoline have shown *in vitro *inhibition of ACTH secretion in corticotroph tumor cells [56-58], suggesting they may be effective in patients with Cushing's disease. Miller and Crapo [42] summarized the results of small studies of patients with Cushing's disease (n = 1-10) treated with bromocriptine. Of 23 patients with Cushing's disease treated with bromocriptine 1.25-30 mg/day for 3-180 weeks, 42% achieved normalization of urine or plasma glucocorticoid levels [42], and ACTH levels decreased by more than 50% in 18% of patients. Subsequent consecutive studies on the effects of bromocriptine in patients with Cushing's disease did not confirm relevant efficacy. In a recent study of 20 patients with persistent Cushing's disease treated with cabergoline 1-3 mg/week post surgery, 7 patients (35%) achieved normalized UFC levels after 3 months of treatment, and an additional 8 (40%) achieved a ≥25% reduction in UFC [59]. After 1 year of cabergoline 1-7 mg/week (median dose 6 mg/week), 50% of patients had normalized UFC, whereas at 2 years at a median dose of 3.5 mg/week, 40% of patients had normalized UFC [59]. These promising results need confirming in larger studies in patients with Cushing's disease.

**Figure 1:**
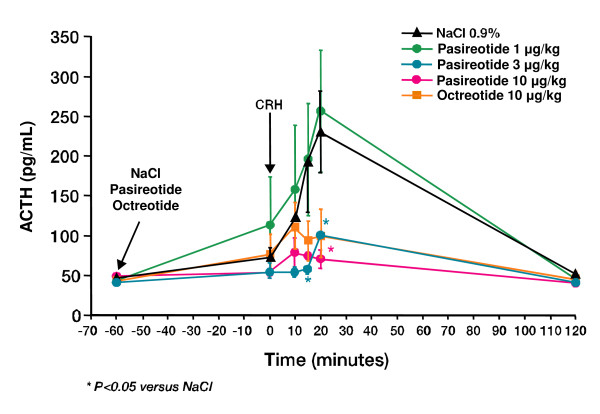
**Regulation of CRH-induced secretion of ACTH in rats by pasireotide **[70]. ^© ^2005 *Eur J Endocrinol. *Reproduced with permission

Human corticotroph adenomas also express multiple somatostatin receptor subtypes (sst receptors), with expression of sst_5 _predominating [60]. Octreotide has high affinity for sst_2 _and moderate affinity for sst_5_, and is mostly ineffective in Cushing's disease [61-63]. This lack of effect in corticotroph cells may be explained by downregulation of sst_2 _receptors [64]. The novel multi-receptor tageted somatostatin analogue pasireotide has high affinity for sst_1_, _2_, _3 _and sst_5_. The functional activity of pasireotide (based on EC_50 _values) on sst_1_, sst_3 _and sst_5 _is >30-fold, 11-fold and 158-fold higher, respectively, than that of octreotide, and is approximately 7-fold lower on sst_2 _[65]. In preclinical studies, pasireotide has shown significant inhibition of basal and stimulated ACTH release in human ACTH-secreting pituitary adenomas [66,67] and in AtT-20 murine corticotroph tumor cells [66,68], as well as significant inhibition of corticotropin-releasing hormone (CRH)-induced ACTH release in rats (Figure 1) [69,70].

**Figure 2:**
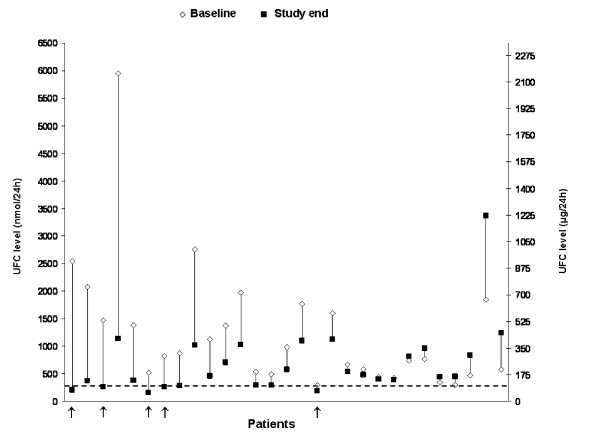
**Phase II study of 29 patients with *de novo *or persistent Cushing's disease receiving pasireotide 600 μg sc bid for 15 days**. Mean UFC level at baseline and study end (day 15) in each patient (n = 29) are shown. The normal range for UFC is 55-276 nmol/24 h (20-100 μg/24 h); the dashed line indicates the upper limit of the normal range. Responding patients (defined as having a UFC level within the normal range at study end) are indicated by the arrows [71]. ^© ^2009 The Endocrine Society. Reproduced with permission.

Results from a short-term, open-label Phase II study of pasireotide showed that it has the potential for being a pituitary-targeted medical treatment for Cushing's disease. Of 29 patients with *de novo *or persistent Cushing's disease who received pasireotide 600 μg sc bid for 15 days, UFC levels decreased in 76% of patients and normalized in 17% of patients (Figure 2) [71]. Adverse events associated with pasireotide were consistent with those observed with other somatostatin analogues, and included predominantly mild gastrointestinal events and mostly transient increases in blood glucose levels. To further determine the role of pasireotide in patients with *de novo*, persistent or recurrent Cushing's disease, a Phase III, randomized (between two doses), double-blind, multicenter trial is ongoing.

The potential for interaction between somatostatin and dopamine receptors to achieve greater suppression of ACTH levels is being explored with the development of chimeric agents. BIM 23A760 is one such agent, with high sst_2 _and D_2 _activity and moderate sst_5 _activity [30,31]. Observation of a high co-expression of sst_5 _and D_2 _in the majority of human corticotroph adenomas studied supports the potential for this agent in the treatment of Cushing's disease; clinical evaluation has not yet been performed [72].

#### Other approaches

Mifepristone is the only available glucocorticoid receptor antagonist. Although clinical data are currently limited in patients with Cushing's disease, early clinical data have demonstrated effective treatment of hypercortisolism, but close monitoring of potentially severe hypokalemia, hypertension, and clinical signs of adrenal insufficiency is required [73,74].

Retinoic acid has been shown to be potentially useful in decreasing corticotroph secretion and proliferation in rodent models, and more recently in a dog model of Cushing's disease [75]. However, the effective dose used in dogs is high and clinical trial results in humans are not currently available.

#### Conclusions

Current adrenal-directed medical treatments for Cushing's disease have significant adverse effects and do not affect the underlying disease or restore normal HPA dynamics. Pituitary-directed medical therapy would instead target the underlying cause of the disease and should be pursued. Although PPAR-γ agonists have demonstrated some efficacy in Cushing's disease, data are limited and there is currently no routine role for these agents. A small, short-term study suggests that cabergoline may be effective in treating a subset of patients with Cushing's disease, but more data are required to address the long-term safety of cabergoline in these patients. Pasireotide has shown promise as a pituitary-directed medical therapy in Cushing's disease in a Phase II study. Long-term efficacy and safety data from Phase III studies will determine whether pasireotide will have a role in the treatment of Cushing's disease.

### Management of acromegaly

Acromegaly results from chronic hypersecretion of growth hormone (GH) from a GH-secreting pituitary adenoma in >90% of patients [76]. GH induces the synthesis of insulin-like growth factor-1 (IGF-1) via hepatic GH receptors; elevated levels of GH and IGF-1 result in metabolic dysfunction and somatic growth. The classic clinical features of acromegaly include acral enlargement, sweating, headaches and glucose intolerance, and chronically unrestrained GH hypersecretion can lead to jaw prognathism, osteoarthritis, frontal bone bossing, diabetes mellitus, hypertension, and respiratory and cardiac failure [77-80]. Compared with the general population, patients with acromegaly have 2.4- to 4.8-fold higher risk of mortality [77,81,82].

The overall goals in the management of acromegaly are to eliminate morbidity and restore the increased mortality rate to normal age- and sex-adjusted rates [81-83]. These goals can be achieved using treatments that remove the tumor mass or reduce and/or control tumor growth, and restore GH secretion to normal. Reducing GH levels has been known to reduce mortality rates for some time [84], and the target GH level has been lowered progressively as more data are acquired. Currently, the criteria for cure of acromegaly are GH levels less than 0.4 μg/L after an oral glucose load using a sensitive GH assay, and IGF-1 levels within the normal age- and sex-adjusted range [83]. Therapeutic goals in acromegaly are to reduce and/or stabilize tumor size, control plasma GH and IGF-1 levels (fasting GH levels <2.5 μg/L and IGF-1 levels within the normal range), preserve pituitary function and prevent recurrences. Treatment modalities for acromegaly include transsphenoidal surgery, medical therapy and radiotherapy.

#### Pituitary surgery

Transsphenoidal surgery is considered the first-line treatment for the initial management of patients with acromegaly, particularly patients with microadenomas who are likely to be cured after surgical resection [85]. Remission rates are highest in patients with microadenomas (75%) and lowest in those with extrasellar macroadenomas; the remission rate in patients with a suprasellar macroadenoma and visual defects is approximately 33% [86]. When the surgery is performed by an experienced and pituitary dedicated neurosurgeon, transsphenoidal surgery has the potential to achieve biochemical cure and is associated with low mortality (<0.1%) and morbidity (<2%). However, approximately 70% of patients harbor macroadenomas; the majority of these patients will have persistent disease following surgery and will require adjuvant medical therapy or radiotherapy [86].

#### Radiotherapy

Conventional external beam radiotherapy has a slow onset of action, taking up to 15 years for maximal disease control, causes hypopituitarism in about 50% of patients and increases cerebrovascular mortality [87]. Stereotactic radiotherapy administered in a single dose (gamma knife, proton beam and LINAC) appears to have a faster onset of action with less hypopituitarism, although long-term data are limited [88,89]. Radiotherapy for persistent disease following surgery normalizes IGF-1 levels in up to 59% of patients depending on the series. By about 5 years after treatment, approximately one-third of patients will have developed pituitary deficiencies, and this is likely to increase with longer follow-up [90,91]. Indications for radiotherapy are post-surgical patients resistant or intolerant to medical therapy, and in recurrent, aggressive tumors.

#### Medical therapy

The three classes of medical therapy for acromegaly are based on receptor targets of the GH/IGF-1 axis: pituitary somatostatin receptor subtypes, pituitary dopamine D_2 _receptors, and peripheral GH receptors [76,85]. Approximately 90% of GH-secreting adenomas express sst_2 _and sst_5_; upon stimulation by a sst ligand, these receptors signal the pituitary to suppress the secretion of GH, resulting in decreased hepatic IGF-1 synthesis. More recently, it has been suggested that a sst ligand may also act peripherally on the GH/IGF-1 axis by binding to somatostatin receptors on peripheral organs, such as hepatocytes in the liver, to inhibit the secretion of IGF-1 [92]. Dopamine agonists bind to pituitary D_2 _receptors, and although the exact mechanism by which dopamine agonists inhibit GH secretion from pituitary adenoma cells is not known, it is thought they act by decreasing the intracellular calcium concentration [85,93]. Thirdly, antagonism of peripheral GH receptors blocks the action of GH at the receptor which inhibits the synthesis of IGF-1 and lowers serum levels; GH levels remain elevated [94], but are blocked by the medication.

Somatostatin analogues are the first choice for the medical treatment of acromegaly in most patients. Octreotide and lanreotide are synthetic analogues of somatostatin that selectively bind to sst_2 _and to a lesser extent sst_5 _(Table 2), and have more prolonged pharmacological actions than the endogenous hormone.

**Table 2:** Somatostatin receptor subtype (sst) binding affinities of somatostatin and analogues in nmol/L [123-126]

	**sst**_**1**_	**sst**_**2**_	**sst**_**3**_	**sst**_**4**_	**sst**_**5**_
Somatostatin-14	0.1-2.3	0.2-1.3	0.3-1.6	0.3-1.8	0.2-0.9
Octreotide	280->1000	0.4-2.1	4.4-34.5	>1000	5.6-32
Lanreotide	180->1000	0.5-1.8	14-107	66->1000	0.6-17
Pasireotide	9.3	1.0	1.5	>1000	0.16

Depot formulations of these analogues, octreotide LAR, lanreotide SR and lanreotide Autogel, have improved the clinical application of these compounds; octreotide LAR and lanreotide Autogel are administered once every 28 days, whereas lanreotide SR is administered once every 7-14 days.

Up to 75% of patients treated for 12-36 months with octreotide LAR as adjuvant therapy achieve control of GH levels and/or IGF-1 levels [95]. In a meta-analysis by Freda *et al*, 57% and 67% of patients treated with octreotide LAR as adjuvant therapy achieved GH levels <2.5 μg/L and IGF-1 normalization, respectively. In patients treated with lanreotide SR as adjuvant therapy, GH levels <2.5 μg/L and IGF-1 normalization was achieved in 48% and 47% of patients, respectively [96]. There are fewer data available for lanreotide Autogel, but it has been shown to be as least effective as lanreotide SR when used as adjuvant therapy in patients with acromegaly [97].

**Figure 3:**
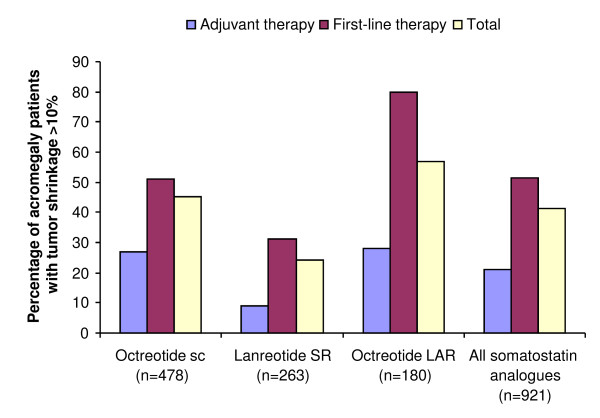
**Effect of somatostatin analogues on pituitary tumor size in patients with acromegaly, showing the percentage of patients with >10% tumor volume reduction after adjuvant or first-line therapy **[107]. ^© ^2005 The Endocrine Society. Reproduced with permission.

First-line therapy with somatostatin analogues in patients with previously untreated acromegaly has received considerable attention since Newman *et al *showed that primary therapy with subcutaneous octreotide was as effective as adjuvant therapy with subcutaneous octreotide (following surgery or radiotherapy) at controlling GH and IGF-1 levels in patients with large or invasive tumors without visual or neurological disturbances [98]. Most studies of first-line therapy of acromegaly with somatostatin analogues evaluated octreotide LAR, and recent studies have demonstrated that first-line therapy with octreotide LAR reduces GH and IGF-1 levels to a similar or greater extent than when administered after surgery and/or radiotherapy (Table 3) [99-106], and reduces pituitary tumor size (usually defined as a 10-25% reduction) in most patients (Table 3 and Figure 3) [99-107]. Indeed, results from a prospective, long-term study (up to 9 years) in 67 patients with *de novo *acromegaly showed that 68.7% and 70.1% of patients treated with octreotide LAR as first-line therapy achieved GH levels <2.5 μg/L and normal IGF-1 levels, respectively, and tumor volume decreased by >25% in 82% of patients [104].

**Table 3:** Summary of results from studies of first-line therapy with octreotide LAR in patients with acromegaly

Reference	No of pts	Duration of treatment	Patients meeting criterion for GH control (%)	Patients with IGF-1 normalization (%)	Mean tumor shrinkage (%)	% of patients with significant tumor shrinkage (definition of significant)
Colao *et al *2001 [101]	15	12-24 months	73	53	53	80 (>20%)
Amato *et al *2002 [99]	8	24 months	50	50	34.8	100 (>10%)
Ayuk *et al *2004 [100]	25	48 weeks	62	64	NR	NR
Jallad *et al *2005 [105]	28	6-24 months	NR	43	NR	76 (>25%)
Colao *et al *2006 [102]	34	6 months	61	45.5	54 (median)	74 (>30%)
Cozzi *et al *2006 [104]	67	6-108 months	69	70	62	82 (>25%)
Mercado *et al *2007 [106]	68	48 weeks	44	34	39	75 (>20%)
Colao *et al *2007 [103]	56	24 months	86	84	68	NR
Colao *et al *2008 [127]	67	12 months	52	58	49	85 (>25%)
Colao et al 2008 [128]	40	48 weeks	NR	NR	35	73 (>20%)

NR = not reported

In order to achieve the reversal of comorbidities and to decrease premature mortality in patients with acromegaly, long-term biochemical control is necessary. Recent studies have demonstrated the importance of regularly monitoring of GH and IGF-1 levels during treatment with somatostatin analogues, and that dose escalation should be performed if optimal control (GH <2.5 μg/L and normal IGF-1 levels) has not been achieved. In the recent study by Colao *et al *[103], in which 56 newly diagnosed patients received octreotide LAR as first-line therapy for 24 months, after 3 months of treatment with octreotide LAR 20 mg/month the dose was increased to 30 mg/month in patients with inadequate control of GH (GH >2.5 μg/L) and/or IGF-1 levels. After a further 9 months, the dose was increased to 40 mg/month in those patients who still remained inadequately controlled. At the end of the 24-month study, 80% of patients had achieved GH and IGF-1 control, and mean GH and IGF-1 levels had decreased by 93% and 63%, respectively. Moreover, 85% of the patients had achieved a tumor volume reduction of >25% (Table 3) [103].

Suitable candidates for first-line therapy with somatostatin analogues might include patients with no risk of visual impairment from the tumor, patients with tumors unlikely to be controlled by surgery (eg lateral cavernous sinus invasion), patients who are at high surgical risk, and patients who decline surgery [83].

Pasireotide is also being evaluated in patients with acromegaly [69,108,109]. Based on the differences in binding affinity and functional activity of pasireotide and octreotide, it can be speculated that in cells and tissues that express sst receptors other than sst_2_, pasireotide may have a stronger inhibitory effect on hormone secretion than octreotide [69]. Moreover, the multi-receptor binding profile of pasireotide suggests that it may be effective in patients with acromegaly resistant or refractory to octreotide. Results from a Phase II study show that pasireotide effectively controls GH and IGF-1 levels in patients with *de novo *or persistent/recurrent acromegaly, and significantly reduces pituitary tumor volume [69,110,111]. Results from an ongoing randomized, double-blind Phase III study comparing the efficacy of a long-acting release formulation of pasireotide with that of octreotide LAR in patients with acromegaly will help determine whether this agent is effective and safe.

Somatostatin analogues are well tolerated in most patients and treatment discontinuations due to adverse events are rare. The most common adverse events associated with somatostatin analogues are injection-site discomfort and erythema, gastrointestinal disturbances such as diarrhea, abdominal pain, flatulence, steatorrhea, nausea and vomiting, biliary sludge or gallstones, and transient hyperglycemia. However, most adverse events are mild or moderate and are transient [95].

Dopamine agonists were the first effective medical therapy for acromegaly, but have since been superseded by somatostatin analogues. Dopamine agonists can control GH and/or IGF-1 levels in 15-40% of patients with acromegaly [112,113]. However, high doses are required and dopamine agonists are associated with nausea, postural hypotension and possible risk of cardiac valve regurgitation [13,14]. The addition of cabergoline to somatostatin analogue therapy may improve response in patients who otherwise are not fully controlled by maximal doses of somatostatin analogues, and in patients with adenomas co-secreting GH and prolactin [114]. The potential for dual somatostatin/dopamine activity in the treatment of patients with acromegaly will be addressed in studies of the chimeric dopamine/somatostatin receptor agonist BIM-23A760. In normal cynomolgus monkeys, BIM-23A760 has been seen to produce potent dose-related GH suppression, with no effect on circulating insulin or glucose levels [115].

Pegvisomant is the only GH receptor antagonist available; it has been shown in clinical trials to normalize IGF-1 levels in up to 97% of patients, and can improve comorbidities such as insulin resistance [94,116-118]. Furthermore, because the efficacy of pegvisomant is not dependent on tumor somatostatin receptor expression, pegvisomant effectively inhibits IGF-1 secretion in patients who are non- or partial responders to somatostatin analogues. GH secretion is not inhibited during pegvisomant therapy and pegvisomant does not treat the tumor, necessitating regular monitoring for pituitary tumor growth [116-118]. Recent studies suggest that the addition of pegvisomant to somatostatin analogue therapy may increase the proportion of patients achieving normal IGF-1 levels, offset the lack of effect of pegvisomant on tumor growth, and improve the quality of life of patients with adequately controlled disease during somatostatin analogue monotherapy [119-121]. Although pegvisomant therapy is generally well tolerated, it is associated with elevated liver enzymes in a small proportion of patients and liver transaminase levels need to be monitored during therapy. Although rare, lipodystrophy at the site of injection has been reported during pegvisomant therapy, suggesting patients should undergo examination of the injection site [122]. Additional studies are required to ascertain the long-term safety of pegvisomant.

## Conclusions

Surgery remains the first-line treatment option in patients with GH-secreting tumors. Somatostatin analogues may be used as first-line therapy in selected patients with large extrasellar tumors not amenable to surgical cure, in patients at high surgical risk, and in patients who decline surgery. Adjuvant therapy with somatostatin analogues is indicated after non-curative surgery and after radiotherapy when GH levels remain elevated. Pegvisomant is typically used in patients who have not responded to surgery and/or radiotherapy and other medical therapies, or in patients in whom glucose control may play a role in the selection of medication (depending on local approval), because it can improve insulin resistance. Dopamine agonists can be considered in patients with a prolactin co-secreting adenoma.

## Summary

▪ First-line treatment of patients with prolactinomas is pharmacotherapy with dopamine agonists; surgery is indicated in the few patients with resistant adenomas who have compromise of the neurovisual apparatus or other adjacent neurological structures, or with contraindications to dopamine agonist use. Radiotherapy is indicated for selected patients, such as those who have not responded to medical or surgical treatment.

▪ First-line treatment of patients with Cushing's disease is pituitary surgery by an expert pituitary surgeon. Results from early, small studies evaluating pituitary-directed medical therapy, such as pasireotide and cabergoline, are encouraging, but further study is needed. Additional approaches include radiation therapy and adrenal-directed therapy, as well as bilateral laparoscopic adrenalectomy for immediate remission of hypercortisolemia when other approaches have failed and patients remain intolerant or incompletely treated.

▪ First-line treatment of patients with acromegaly is pituitary surgery when the outcome of the surgery is likely to be complete resection of the pituitary adenoma, or to debulk significant tumor, particularly if neurological structures are in jeopardy. Somatostatin analogues may be used as first-line therapy for acromegaly in selected patients with large extrasellar tumors not amenable to surgical cure, frail patients, patients at high surgical risk, and patients who decline surgery.

## Competing interests

MB declares no competing interests. VB has research studies ongoing with support from Novartis, and speaker honoraria also from Novartis. BMKB has received research grants and/or consulting honoraria from Ipsen, Novartis and Pfizer. AC has received unrestricted grants by Ipsen, Italfarmaco, Novartis, and Pfizer for research programs in acromegaly, received lectures fees by Ipsen, Italfarmaco, Novartis, and Pfizer and has been a member of the Scientific Boards of Novartis and Ipsen. SP has received consulting fees from Novartis, and lecture honoraria from Novartis and Ipsen.

## Authors' contributions

BMKB, AC, SP, VSB and MB all participated in an independent CME symposium at the 89th Annual Meeting of the Endocrine Society. This manuscript was developed directly from those presentations, which each author prepared him/herself. BMKB and SP directed the development of the manuscript, based on the content of the symposium presentations, with editorial assistance from Keri Wellington. All authors reviewed the manuscript and provided critical input. All authors read and approved the final manuscript.

## Pre-publication history

The pre-publication history for this paper can be accessed here:

http://www.biomedcentral.com/1472-6823/10/10/prepub
